# Predictive value of intratumoral-metabolic heterogeneity derived from ^18^F-FDG PET/CT in distinguishing microsatellite instability status of colorectal carcinoma

**DOI:** 10.3389/fonc.2023.1065744

**Published:** 2023-04-27

**Authors:** Li Zhang, Yu Liu, Ying Ding, Yinqian Deng, Huanyu Chen, Fan Hu, Jun Fan, Xiaoli Lan, Wei Cao

**Affiliations:** ^1^ Department of Nuclear Medicine, Union Hospital, Tongji Medical College, Huazhong University of Science and Technology, Wuhan, China; ^2^ Hubei Key Laboratory of Molecular Imaging, Wuhan, China; ^3^ Department of Pathology, Union Hospital, Tongji Medical College, Huazhong University of Science and Technology, Wuhan, China

**Keywords:** microsatellite instability, positron emission tomography/computed tomography, metabolic parameter, heterogeneity, colorectal carcinoma, immune-checkpoint inhibitors

## Abstract

**Purpose/background:**

Microsatellite instability (MSI) status is a significant biomarker for the response to immune checkpoint inhibitors, response to 5-fluorouracil-based adjuvant chemotherapy, and prognosis in colorectal carcinoma (CRC). This study investigated the predictive value of intratumoral-metabolic heterogeneity (IMH) and conventional metabolic parameters derived from ^18^F-FDG PET/CT for MSI in patients with stage I–III CRC.

**Methods:**

This study was a retrospective analysis of 152 CRC patients with pathologically proven MSI who underwent ^18^F-FDG PET/CT examination from January 2016 to May 2022. Intratumoral-metabolic heterogeneity (including heterogeneity index [HI] and heterogeneity factor [HF]) and conventional metabolic parameters (standardized uptake value [SUV], metabolic tumor volume [MTV], and total lesion glycolysis [TLG]) of the primary lesions were determined. MTV and SUV_mean_ were calculated on the basis of the percentage threshold of SUVs at 30%–70%. TLG, HI, and HF were obtained on the basis of the above corresponding thresholds. MSI was determined by immunohistochemical evaluation. Differences in clinicopathologic and various metabolic parameters between MSI-High (MSI-H) and microsatellite stability (MSS) groups were assessed. Potential risk factors for MSI were assessed by logistic regression analyses and used for construction of the mathematical model. Area under the curve (AUC) were used to evaluate the predictive ability of factors for MSI.

**Results:**

This study included 88 patients with CRC in stages I–III, including 19 (21.6%) patients with MSI-H and 69 (78.4%) patients with MSS. Poor differentiation, mucinous component, and various metabolic parameters including MTV_30%_, MTV_40%_, MTV_50%_, and MTV_60%_, as well as HI_50%_, HI_60%_, HI_70%_, and HF in the MSI-H group were significantly higher than those in the MSS group (all *P* < 0.05). In multivariate logistic regression analyses, post-standardized HI_60%_ by Z-score (*P* = 0.037, OR: 2.107) and mucinous component (*P* < 0.001, OR:11.394) were independently correlated with MSI. AUC of HI_60%_ and our model of the HI_60%_ + mucinous component was 0.685 and 0.850, respectively (*P* = 0.019), and the AUC of HI_30%_ in predicting the mucinous component was 0.663.

**Conclusions:**

Intratumoral-metabolic heterogeneity derived from ^18^F-FDG PET/CT was higher in MSI-H CRC and predicted MSI in stage I–III CRC patients preoperatively. HI_60%_ and mucinous component were independent risk factors for MSI. These findings provide new methods to predict the MSI and mucinous component for patients with CRC.

## Introduction

The world’s second leading cause of cancer death is colorectal cancer (CRC), which is the third most common cancer (1). While surgery and adjuvant chemotherapy are currently the main treatments for CRC, immunotherapy has emerged as a new therapeutic strategy for cancer treatment. The programmed cell death 1 (PD-1)/programmed death-ligand 1 (PD-L1) axis is a critical immune response. Anti-PD-1/anti-PD-L1 blockade therapy enhances the antitumor activity of T cells by blocking the binding of PD-L1 on tumor cells and PD-1 on immune T cells, representing a key anticancer strategy. Blocking these pathways effectively reduces tumor growth and improves survival in most solid tumors (2), especially in microsatellite instability-high (MSI-H) CRC (3). Nivolumab and pembrolizumab, PD-1 inhibitors, were officially approved by the FDA in 2017 for the treatment of MSI-H solid tumors (4), ushering in a new era of immunotherapy.

Under proficient mismatch repair (pMMR) conditions, errors in the replication of microsatellites are repaired by MMR proteins. Mismatch repair deficiency (MMR-D) can lead to errors beyond repair normally and has been detected in endometrial cancer, CRC, and gastric cancer (5). MSI results from errors in the DNA replication of microsatellites and is observed in approximately 12%–15% of localized CRC cases and 4% of stage IV CRC cases (2, 6). The accumulation of MSI eventually leads to development of MSI-H. MSI status has been regarded as an important prognostic biomarker for patients with CRC and affects the selection of adjuvant 5‐fluorouracil-based chemotherapy or immune checkpoint inhibitors. Studies showed that 5-fluorouracil-based adjuvant chemotherapy had limited therapeutic effect for patients with MSI-H CRC (7, 8). MSI tumors have large proportions of mutant neoantigens and tumor-infiltrating lymphocytes (TILs) (2, 6, 9) and a high tumor mutational burden (TMB) that promotes the infiltration of immune cells; therefore, MSI plays a significant role in long-term durable immunotherapy (10, 11).

CRC, especially CRC with MSI-H, exhibits significant heterogeneity, which refers to the different characteristics within a tumor in regard to genomics, histopathologic features, tumor microenvironment, T-cell infiltrate, and response to therapies (9, 12, 13). MSI is generally determined by evaluation of MMR proteins including MLH1, PMS2, MSH2, and MSH6 using immunohistochemical (IHC) or by PCR testing and next-generation sequencing to test MMR genes. However, the above methods are usually invasive and may be affected by intratumoral heterogeneity. Therefore, it is meaningful to screen tumors for MSI status to avoid unnecessary inspections and promote the immunotherapy of early-stage CRC patients.

PET/CT is a systemic examination that can detect the distant metastasis of tumors. In recent years, ^18^F-FDG PET/CT has demonstrated efficiency in staging, predicting prognosis, assessing treatment response, and determining gene mutational status in patients with CRC (14–17). MSI-H CRCs have higher intratumoral heterogeneity compared with microsatellite stability (MSS) CRCs (9, 12, 13, 18). ^18^F-FDG PET/CT can reflect the intratumoral-metabolic heterogeneity (IMH) by the heterogeneity index (HI) and the heterogeneity factor (HF). These two quantitative indicators reflect IMH and have already been shown to predict survival outcomes in epithelial ovarian cancer, gastric cancer, and oral cavity squamous cell carcinoma (19–21). However, the role of IMH derived from ^18^F-FDG PET/CT in predicting MSI in CRC patients is unknown (22, 23). Therefore, we aimed to explore the ability of IMH and conventional metabolic parameters (standardized uptake value [SUV], metabolic tumor volume [MTV], and total lesion glycolysis [TLG]) derived from ^18^F-FDG PET/CT for the prediction of MSI status in patients with stage I–III CRC.

## Materials and methods

### Patient inclusion and exclusion

This study included patients who underwent ^18^F-FDG-PET/CT in Wuhan Union Hospital from January 2016 to May 2022. The inclusion criteria were as follows: patients with (a) colorectal lesions diagnosed as colorectal adenocarcinoma with or without a mucinous component (part of the tumor volume with mucin associated likely produced by malignant glands or forming mucin pools, which account for 5%–100% of the tumor volume) by pathology; (b) complete ^18^F-FDG PET/CT images before surgery within 1 month of surgery; and (c) IHC staining evaluated MMR protein expression status.

**Figure 1:**
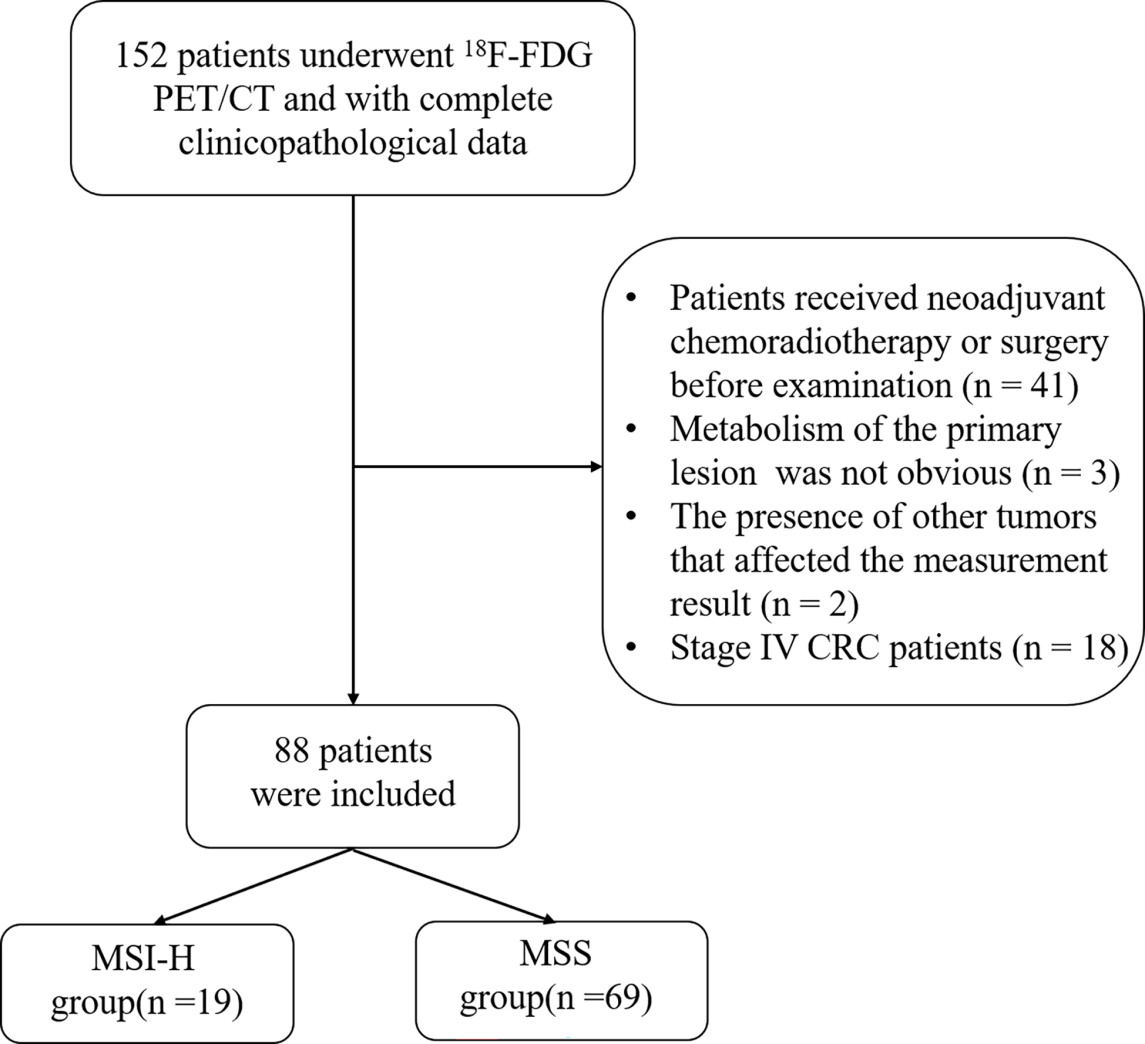
Flowchart of patient selection.

The exclusion criteria were as follows: (a) patients who received neoadjuvant chemoradiotherapy or surgery before ^18^F-FDG PET/CT examination (n = 41); (b) the primary lesion did not show metabolism on PET/CT (n = 3); (c) the presence of other tumors that interfered with the measurement of the CRC primary tumor (n = 2); and (d) CRC patients in stage IV (n = 18). The flowchart is shown in **Figure 1**.

### PET/CT imaging acquisition protocol

All patients got whole-body imaging (from the cranium to the upper third of the thighs) by ^18^F-FDG PET/CT scans with a dedicated PET/CT system (GE Healthcare, Milwaukee, WI, USA; Discovery VCT and Discovery LS). An intravenous injection of 3.7–4.4 MBq/kg ^18^F-FDG was administered, after at least 6 h of fasting and a blood glucose level drops below 200 mg/dl. The examination was performed approximately 60 min after the injection of ^18^F-FDG. A slice spiral CT scan was first performed. The PET imaging was collected in three‐dimensional mode at 2 min per frame for a total of seven to eight frames. PET data were reconstructed on the basis of ordered-subset expectation maximization. The reconstructed images were processed on a workstation (Xeleris Workstation, GE Healthcare).

### Measurement of PET metabolic parameters

**Figure 2:**
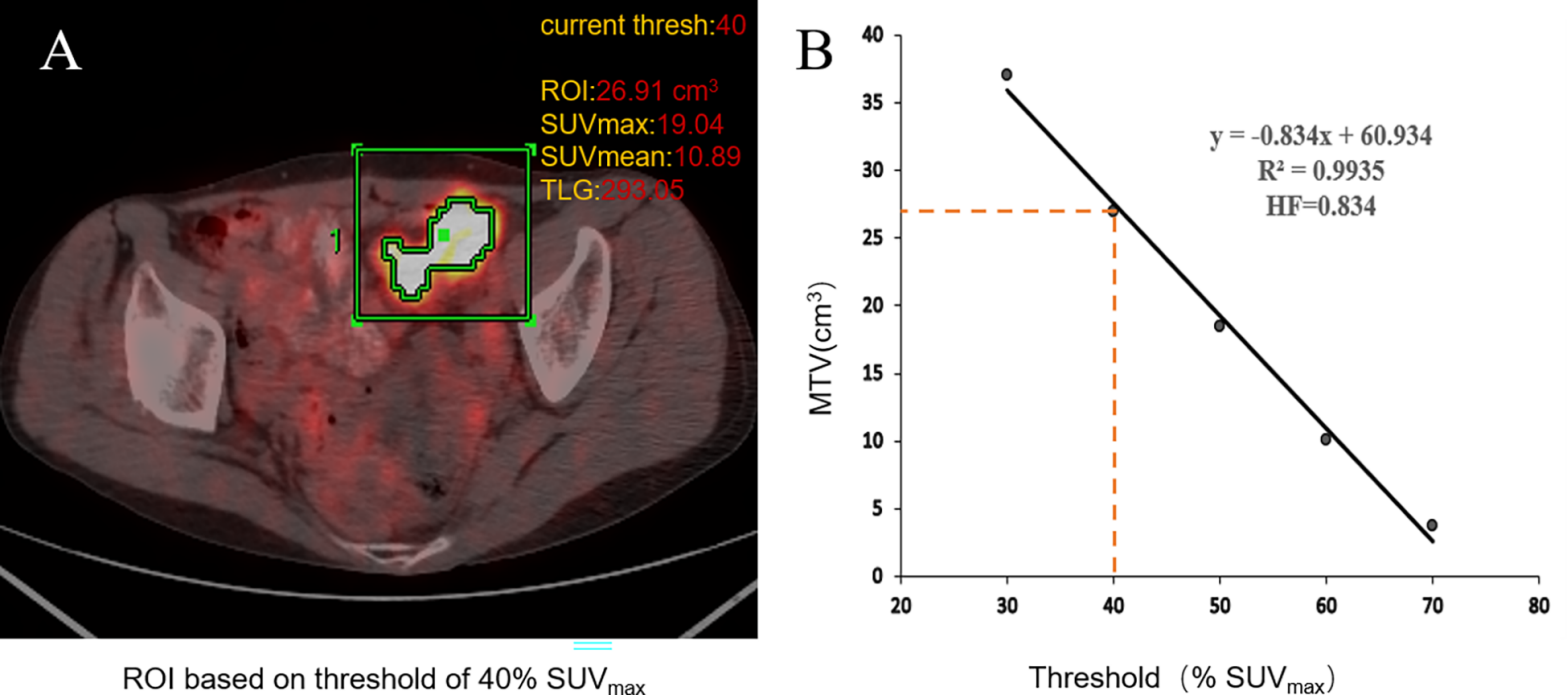
Workflow of ^18^F-FDG PET/CT metabolic parameter measurements. When using the percent threshold of 40%, SUV_max_ was 19.04, the volume of ROI was 26.91 cm^3^, SUV_mean_ was 10.98, and TLG was 293.05. HI = SUV_max_/SUV_mean_ = 1.75 **(A)**. The HF is the absolute value of the linear regression slope calculated by the least square method from the different threshold of the ROI; the threshold of tumor volumes ranged from 30% to 70% of SUV_max_ **(B)**.

The PET/CT image was evaluated in a blind manner by two nuclear medicine doctors, each with more than 6 years of clinical experience. The ROI of the primary lesions of CRC patients was delineated in the workstation. The quadrate working frame was placed on the primary lesion, and if necessary, areas of physiologic uptake or nearby lymph node metastasis were manually excluded. The SUV_max_ of the primary lesion was measured automatically by the workstation (AW4.6; GE Healthcare). The SUV_mean_ and MTV were obtained by the percent threshold of SUV_max_ in the working frame. MTV_30%–70%_ were calculated with 30%–70% of SUV_max_ as the percent threshold, respectively (23). SUV_mean_ was obtained from the corresponding MTV. TLG = MTV × SUV_mean_. IMH contains the HI and HF. HI = SUV_max_/SUV_mean_. HI_30%–70%_ was determined from the primary tumors. In addition, the HF was the absolute value of the linear regression slope calculated by the least square method from the different threshold of the ROI (**Figure 2**) (21).

### Immunohistochemical evaluation

Clinicopathological data, including differentiation grade, mucinous component, TN stages, tumor deposit, lymphovascular invasion, perineural invasion, and IHC results for four MMR proteins (MLH1, MSH2, MSH6, PMS2), were evaluated by pathologists. The absence of any MMR protein was defined as MMR-D, also known as MSI-H. The expression of all four MMR proteins was defined as pMMR, characterized as MSS. All patients in stage I–III CRC had undergone surgery within 1 month after the PET/CT scan.

### Statistical analysis

The quantitative variable distribution was analyzed between groups. Following the distribution of data, data were expressed by mean ± standard deviation or median (interquartile range) and analyzed by T-test or Mann–Whitney U test, respectively. The categorical data were examined using the Fisher’s exact test. Various metabolic parameters were standardized by Z-score normalization before performing logistic regression analysis, as the significant differences in various metabolic parameter values. The mathematical model was built, as described in the study by Wang et al. (24). The ROC curve was used to estimate the predictive value of different metabolic parameters for MSI. The DeLong test was used to assess the variation in AUC values. All data were analyzed by SPSS software version 23 (IBM Corp, Armonk, NY, USA) and MedCalc software (version 19.7.2). Statistical significance was defined as a two-sided *P* value less than 0.05.

## Results

### Patient characteristics

This study included 88 patients, including 59 male patients (70.2%) and 29 female patients (29.8%). The average age was 65 years (range 28–89 years). Among the total 88 patients, 19 (21.6%) patients were categorized into the MSI-H group and 69 (78.4%) patients were categorized into the MSS group. Patients and tumor characteristics are summarized in **Table 1**. The MSI-H CRC group tended to have a mucinous component, poor differentiation, and a larger maximum tumor diameter, with a higher frequency of male patients compared with the MSS CRC group, with statistical significance (all *P* < 0.05). Age, primary tumor location, TN stage, AJCC-TNM stage, tumor deposit, lymphovascular invasion, and perineural invasion were not significantly different between the MSI-H group and the MSS group (all *P* > 0.05).

**Table 1:** Clinicopathologic characteristics of patients.

Characteristics	Number	MSI-H (n = 19)	MSS (n = 69)	*P* value
Age				0.282^*^
<60	28	8	20	
≥60	60	11	49	
Gender				0.026^*^
Male	59	17	42	
Female	29	2	27	
Primary tumor location				0.405^*^
Left	35	6	29	
Right	34	10	24	
Rectum	19	3	16	
Differentiation				0.019^*^
Poor	12	6	6	
Well or moderate	76	13	63	
Mucinous component				<0.001^*^
Positive	16	10	6	
Negative	72	9	63	
Maximum tumor diameter (cm)		4.98±1.29	4.19±1.73	0.037**^#^**
T stage				1^*^
T1-T2	12	2	10	
T3-T4	76	17	59	
N stage				0.276^*^
0	59	15	44	
1-2	29	4	25	
AJCC-TNM stage				0.328^*^
I	10	2	8	
II	49	13	36	
III	29	4	25	
Tumor deposit				0.11^*^
Negative	78	19	59	
Positive	10	0	10	
Lymphovascular invasion				1^*^
Negative	68	15	53	
Positive	20	4	16	
Perineural invasion				0.138^*^
Negative	66	17	49	
Positive	22	2	20	

MSI, microsatellite instability; MSS, microsatellite stability; AJCC, American Joint Committee on Cancer; TNM, tumor–node–metastasis. P values were calculated using the *Fisher’s exact test and ^#^T-test.

### Differences in PET/CT metabolic parameters between the MSI-H group and the MSS group

In terms of conventional metabolic parameters, the MSI-H group tended to have higher MTV_30%_, MTV_40%_, MTV_50%_, and MTV_60%_ compared with the MSS group, with significant differences (all *P* < 0.05). Regarding IMH, HI_60%_, HI_70%_, and HF were significantly higher (all *P* < 0.05) in the MSI-H group compared with the MSS group. No significant difference was found in SUV_max_ and other metabolic parameters between the groups. Details of IMH and conventional metabolic parameters in the MSS group and MSI-H group are listed in **Table S1**.

### Independent predictive indicators of PET/CT parameters and clinical parameters for MSI

The aforementioned significant characteristics were included into logistic analysis. Univariate logistic analysis showed that the expression of MSI in CRC was associated with mucinous component, differentiation, and standardized parameters Z-HI_50%_ and Z-HI_60%_ (all *P* < 0.05) (**Table 2**). Multivariate logistic regression analysis showed that mucinous component and Z-HI_60%_ were the independent predictive indicators for MSI of the CRC (**Table 3**).

**Table 2:** Univariate regression analyses for predicting MSI in CRC patients.

Factors	*P* value	Odds ratio	95% CI
Differentiation	0.016	0.206	0.06-0.74
Mucinous component	<0.001	11.70	3.41-39.90
Maximum tumor diameter (cm)	0.074	1.332	0.97-1.83
Z-MTV_30%_	0.136	1.404	0.90-2.19
Z-MTV_40%_	0.150	1.384	0.89-2.15
Z-MTV_50%_	0.173	1.360	0.87-2.12
Z-MTV_60%_	0.221	1.316	0.85-2.04
Z-HI_50%_	0.039	1.747	1.03-2.98
Z-HI_60%_	0.042	2.105	1.03-4.31
Z-HI_70%_	0.059	2.590	0.96-6.97
Z-HF	0.133	1.414	0.90-2.22

Z-, processed by Z-score standardization method; MTV, metabolic tumor volume; TLG, total lesion glycolysis; HI, heterogeneity index; HF, heterogeneity factor; CI, confidence interval.

**Table 3:** Multivariate regression analyses for predicting MSI in CRC.

Factors	*P* value	Odds ratio	95% CI	*P* value	Odds ratio	95% CI
Differentiation	0.090	0.260	0.06-1.24	0.113	0.278	0.06-1.35
Mucinous component	0.001	9.348	2.53-34.51	<0.001	11.394	2.96-43.89
Z-HI_50%_	0.057	1.717	0.98-3.0			
Z-HI_60%_				0.037	2.107	1.05-4.25

Z-, processed by Z-score standardization method; HI, heterogeneity index; CI, confidence interval.

### Model establishment and mucinous composition exploration

**Figure 3:**
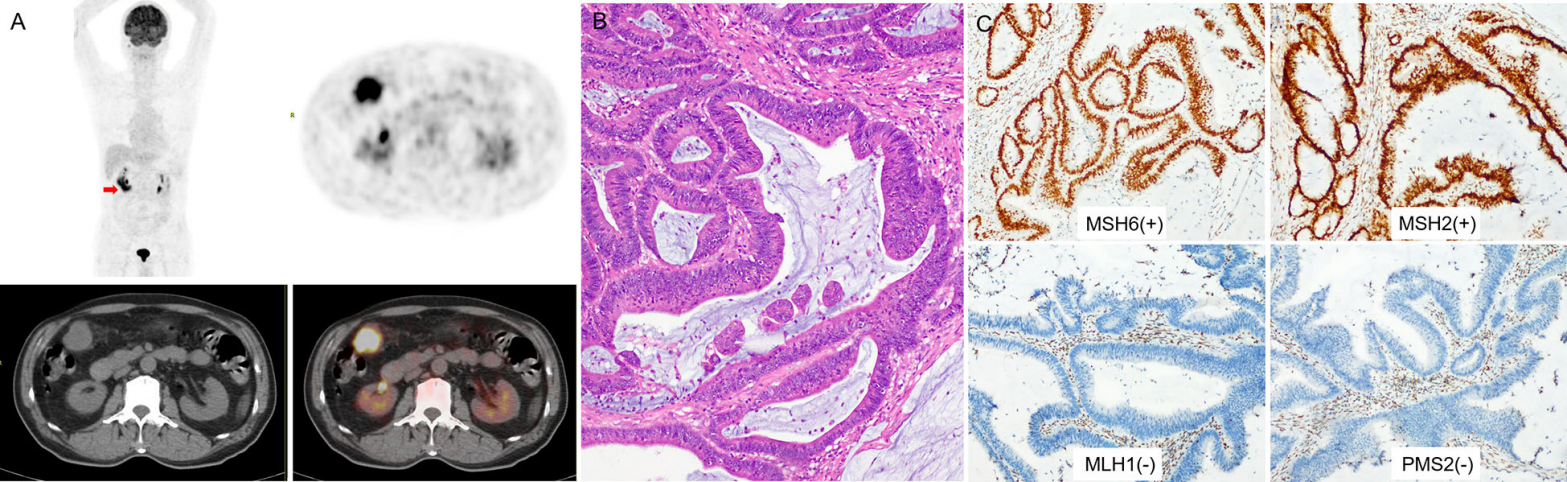
**(A)** a 73-year-old man with right colon cancer. PET/CT showed high ^18^F-FDG accumulate in the colon of hepatic flexure (arrow; SUV_max_ 10.8; HI_60%_ 1.43). **(B)** Pathological analysis revealed well-differentiated carcinoma; in H&E, the bulk of the malignant glands produced visible mucin. **(C)** IHC analysis demonstrated MSI-H, MMR proteins showed as MSH6(+), MSH2(+), MLH1(-), PMS2(-).

**Figure 4:**
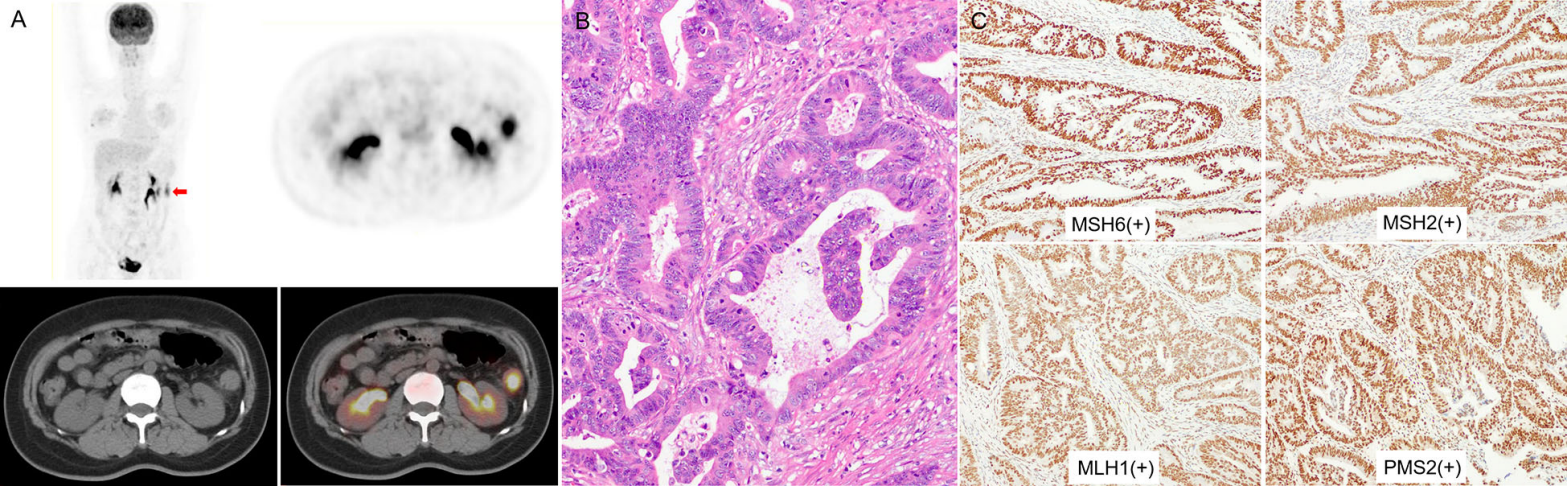
**(A)** a 48-year-old woman with left colon cancer. ^18^F-FDG PET/CT showed hypermetabolism in the descending colon (arrow; SUV_max_ 9.86; HI_60%_ 1.38). **(B)** The pathological diagnosis was moderately differentiated CRC, without mucinous component. **(C)** IHC analysis results for MMR proteins were MSH6(+), MSH2(+), MLH1(+), and PMS2(+), indicating MSS.

**Figure 5:**
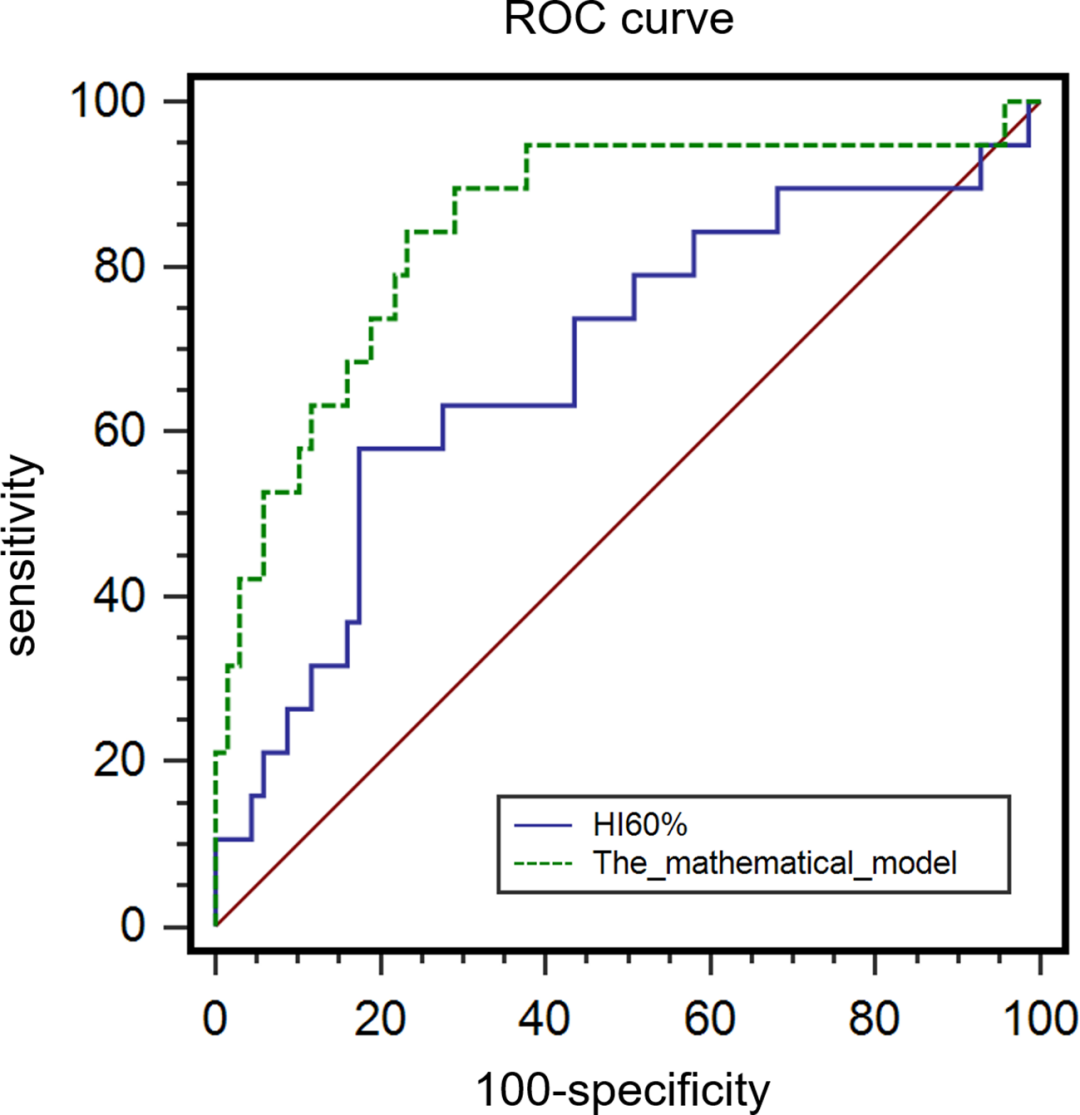
ROC curve for HI_60%_ and the mathematical model in predicting MSI.

MSI-H CRC had higher IMH and MTV values in preoperative PET/CT examination and were more prone to exhibit a mucinous component than MSS CRC (**Figures 3**, **4**). A model of the HI_60%_+ mucinous component was established, and the formula was as follows: y = exp(x)/[1+exp(x)], x = -2.083 + 0.749 × Z-HI_60%_+ 2.589 × mucinous component (**Table 4**) (the “-” or “+” of mucinous component was defined as “0” or “l”). (**Table S3**) The cutoff value determined by ROC analysis was y = 0.1432, when y > 0.1432 was considered as MSI-H CRC, while y < 0.1432 was considered as MSS CRC. The AUC of HI_60%_ and the mathematical model were 0.685 and 0.85, with a sensitivity of 0.579 and 0.842 and specificity of 0.826 and 0.768, respectively. There was a significant difference in AUC between HI_60%_ and our model (Z = 2.339, *P* = 0.019). The predictive ability of ROC curves is illustrated in **Figure 5**.

The Mann–Whitney U test was used to further evaluate the connection between mucinous components and various metabolic parameters. HI_30%_ was significantly higher in CRC with mucinous component than in CRC without mucinous component. The AUC of HI_30%_ was 0.663, with sensitivity of 0.875 and specificity 0.5, respectively. The cutoff value was 1.974. The performance of PET/CT parameters is illustrated in **Table S2**.

## Discussion

Our study examined the value of ^18^F-FDG PET/CT imaging parameters in identifying MSI in stage I–III CRC and explored the intratumoral heterogeneity. As we know, this is the first study examining the utility of IMH determined by PET/CT to predict MSI of CRC. Our results showed that HI_60%_ and the mucinous component were independent prediction factors for MSI status in stage I–III CRC.

CRC is tumor with high glucose metabolism that rapidly converts glucose to lactate (known as the “Warburg effect”) (25, 26). Other effects include m6A modifications (27), glycolysis-associated lncRNA of colorectal cancer (GLCC1) (28), and insulin-like growth factor 1 (IGF-1) signaling (29), which could also enhance glycolysis in different paths. SUV_max_, as a semiquantitative parameter in the ^18^F-FDG uptake of lesions, is considered to be an auxiliary index for judging benign and malignant tumors and a general prognostic factor in malignancies (30). The limitation of SUV_max_ is that it only reflects a single voxel value and cannot reflect the characteristics of the entire ROI. MTV is an indicator usually based on a fixed SUV threshold of 2.5 or percentage SUV_max_ threshold of 40%–42% (30), reflecting the tumor burden of the tumor; it plays an important role in predicting prognosis (30) and even target volume delineation before radiotherapy (31). TLG can reflect both volume and metabolic activity of lesions, which can reflect the advantage of PET/CT better. While SUV_max_, MTV, and TLG are established as parameters to assess for diagnosis, efficacy, prognosis, tumor burden, and TMB in cancer (32, 33), they have limited value in judging IMH.

IMH is one of the characteristics of solid tumors. The measurement of tumor heterogeneity is of great significance to the drug resistance, tumor invasion, and prognosis of patients (33, 34). The difference in proliferation, receptor expression, necrosis, hypoxia, energy metabolites, micro-vessel density, protein expression, and gene expression in tumors may lead to uncertainties in therapeutic effects (9, 18, 35, 36). ^18^F-FDG PET/CT can reflect tumor metabolic heterogeneity through different parameters, including HI (21, 33), HF (19), coefficient of variation (19, 20, 33), and texture parameters (34). In our research, HI was calculated by dividing SUV_max_ by SUV_mean_ for the tumor, which reflects not only the ^18^F-FDG metabolism in the ROI of tumors but also the average of metabolic activity. Research showed that higher HI_30%_ levels were related to poorer overall survival in patients with oral cavity squamous cell carcinoma (21). In the study by Kim et al. (37), a lower HI based on a threshold of 2.5 of the primary tumors was associated with better prognosis in locally advanced nasopharyngeal carcinoma. Compared with calculation of the HI, calculation of the HF is more complicated and determined by linear regressions of MTVs under different SUV thresholds, determined by two methods (percentage threshold method and fixed threshold method). In general, the percentage threshold method is suitable for lesions with high SUV_max_, which avoids interference with the surrounding normal tissue metabolism (21). The fixed threshold method is more suitable for tumors with a low ^18^F-FDG uptake (38). For this reason, we selected the percentage threshold method in our study. In a study of 55 patients with advanced gastric cancer (19), HF (SUV threshold based on 2.5–3.5) was significantly associated with TNM stage and overall survival.

In our study, several conventional metabolic parameters were significant predictors of MSI, which was similar to previous findings (23, 39). Song et al. (39) retrospectively analyzed the relationship between conventional metabolic parameters based on the threshold of 40% SUV_max_ in 420 CRC patients. The results proved that MTV ≥32.19 cm^3^ of CRC was linked to the presence of MSI-H and the increased density of TILs in MSI-H CRC may lead to higher TMB. The result was consistent with the previous study of Liu et al. (23) and our results (**Table 4**). Moreover, our analysis further proved the existence of IMH of CRC. The HI is the ratio of the highest ^18^F-FDG uptake and the average ^18^F-FDG uptake of the lesions. MSI-H CRCs showed a higher HI compared with MSS CRC even though SUV_max_ and SUV_mean_ were not statistically different in the two groups, which might be caused by the larger proportion of mutant neoantigens increasing glucose metabolism and upregulated immune checkpoints of MSI-H CRC that preserve MSI-H CRC from the pernicious immune microenvironment (2). Therefore, HI_60%_ could make up for the limitation of conventional metabolic parameters in predicting MSI. HF reflects the metabolic changes in tumors under different thresholds. In our research, MSI-H CRC had a higher HF than MSS CRC, which indicated a higher heterogeneity in MSI-H CRC.

**Table 4:** PET/CT for predicting MSI.

	Sensitivity	Specificity	AUC	Stage (I–IV)	Sample size (MSI-H/MSS)	Location
HI_60%_ our model	0.579 0.842	0.826 0.768	0.658 0.850	I–III	88 (19/69)	Colorectal
Li’s model (22)	0.833	0.763	0.828	I–IV	173 (13/160)	Colorectal
Liu’s result (23)	0.929	0.667	0.805	I–IV	44 (14/30)	Colorectal
Song’s result (39)	0.523	0.766	0.633	II–IV	420 (44/376)	Colorectal

Related parameters: our model: HI_60%_ and mucinous component; Li’s model: CEA, one PET feature (wavelet-LHH_firstorder_Skewness_PET) and one CT feature (wavelet-HHL_firstorder_RootMeanSquared_CT); Liu’s result: MTV_50%_; Song’s result: age, primary lesion located, and MTV_40%_.

MSI-H CRC is a tumor with higher heterogeneity and increased TMB and tumor burden compared with MSS CRC. MSI-H CRC has more TILs in both tumor and tumor-adjacent tissues. The higher CD3+ and CD8 T lymphocyte densities are associated with higher tumor size and MTV value (9, 12, 39). The consumption of glucose by tumor limits T-cell metabolism, leading to the dampened glycolytic capacity, mTOR activity, and IFN-γ production, representing the adaptation of resistance to the MSI-induced immunoreactive microenvironment and thereby allowing tumor progression. Due to the inhibition of TIL metabolism, the insignificant change of SUV_mean_ in HI_60%_ can be explanted. Checkpoint blockade antibodies against PD-1, PD-L1, and CTLA-4 can restore glycolysis, IFN-γ production, and immune responses in T cells (36, 40) and lead to an insignificant decrease of SUV_mean_ in MSI-H solid tumors in the early stage of immunotherapy.

Mucins are highly O-glycosylated glycoproteins that are essential for a variety of biological processes. Tumor growth results in an unfavorable microenvironment; however, mucins help to evade acidity, hypoxia, and other inhospitable conditions that block drug delivery and promote cancer progression. Mucin is classified into more than 20 subtypes, and the functions are not fully understood (41). MUC2 and MUC5AC mucins produced by colonic goblet cells tend to overexpress in mucinous CRC and MSI-H CRC (42). The EGFR pathway substantially leads to the formation of CRC with the mucinous component, regardless of the percentage (43). Additionally, the mutation rate of RAS/RAF/MAPK and PI3K/AKT pathways in mucinous adenocarcinoma (with more than 50% of mucinous component) is higher than that in non-mucinous adenocarcinoma (42). Our study revealed a higher HI_30%_ in CRC with a mucinous component than in CRC without a mucinous component. Therefore, ^18^F-FDG PET/CT may contribute to detect the existence of a mucinous component in CRC and help to predict MSI of CRC.

Multivariate logistic regression analysis demonstrated that Z-HI_60%_ and mucinous component were independent risk factors of MSI in stage I–III CRC. These results indicate that more attention should be given to heterogeneity of tumors in the early stage. MSI-H in CRC is associated with pathological features such as mucinous carcinoma, proximal colon, poorly differentiation, lymphatic invasion, tumor staging, tumor size, KRAS mutation, and BRAFV600E mutation (2, 6); some results of our study are consistent with these findings. CRC with MSI-H more frequently appears in stage II cases (approximately 20%) compared with stage III (approximately 12%) and is relatively uncommon among stage IV CRC (around 4%) (6). Compared with pMMR/MSI tumors, stage I–III CRC with MSI-H generally have a better prognosis. Stage IV CRC has significant heterogeneity compared with stage I–III CRC, especially between the metastasis and primary tumors (35). The assessment of M staging is one of the most obvious advantages of PET/CT examination. Early detection of intratumoral heterogeneity may be helpful for the formulation of clinical treatment decisions; to avoid the influence of the differences of intertumoral heterogeneity, we selected stage I–III CRC into our research.

The proportion of MSI-H in our study (21.6%) was higher than that in epidemiological reports (12%–15%), which may be because MSI-H CRC is more common in hypermetabolic CRC and makes it easier to be found by PET/CT. In addition, our research object was stage I–III CRC, while MSI-H CRC accounts for only 4% of stage IV CRC (6), which implied that many MSS CRC patients were not included in this study. Although we had built a mathematical model, the limitations of our study include the limited number of cases and the absence of a validation cohort to support, and thus further data validation is required. As a retrospective study, selective bias was inevitable. In our research, imaging data were obtained from two different equipment. While monthly quality control was routinely performed and the same workstation was used, there may still be some uncontrollable differences in the measurement of metabolic parameters by the two devices. Finally, our research was based on a single center, and therefore data from more centers need to be included to validate the utility of IMH and conventional metabolic parameters for predicting MSI in patients with CRC.

## Conclusion

This study not only demonstrated that MSI-H CRCs have a higher tumor metabolic burden than MSS CRCs but also revealed a higher IMH in MSI-H CRCs compared with MSS CRCs. The HI_60%_ derived from ^18^F-FDG PET/CT and mucinous component were the independent risk factors for MSI in CRC. The mathematical model from HI_60%_+ mucinous component demonstrated the highest predictive performance. PET/CT is a non-invasive approach to evaluate MSI and the mucinous component in CRC and will help in guiding immunotherapy in CRC patients.

## Data availability statement

The raw data supporting the conclusions of this article will be made available by the authors, without undue reservation.

## Ethics statement

The studies involving human participants were reviewed and approved by the Institutional Review Board of Union Hospital, Tongji Medical College, Huazhong University of Science and Technology. Written informed consent from the participants’ legal guardian/next of kin was not required to participate in this study in accordance with the national legislation and the institutional requirements. Patient consent was waived due to the retrospective design.

## Author contributions

Conceptualization, LZ, YD, and WC; formal analysis, WC; investigation, YL, YQD, and HC; resources, XL, JF, WC, and FH.; writing—original draft preparation, LZ; writing—review and editing, LZ, YL, YQD, and HC; funding acquisition, WC. All authors contributed to the article and approved the submitted version.
