# Corrigendum: Predictive value of intratumoral-metabolic heterogeneity derived from ^18^F-FDG PET/CT in distinguishing microsatellite instability status of colorectal carcinoma

**DOI:** 10.3389/fonc.2023.1351842

**Published:** 2024-01-05

**Authors:** Li Zhang, Yu Liu, Ying Ding, Yinqian Deng, Huanyu Chen, Fan Hu, Jun Fan, Xiaoli Lan, Wei Cao

**Affiliations:** ^1^ Department of Nuclear Medicine, Union Hospital, Tongji Medical College, Huazhong University of Science and Technology, Wuhan, China; ^2^ Hubei Key Laboratory of Molecular Imaging, Wuhan, China; ^3^ Department of Pathology, Union Hospital, Tongji Medical College, Huazhong University of Science and Technology, Wuhan, China

**Keywords:** colorectal carcinoma, heterogeneity, immune-checkpoint inhibitors, metabolic parameter, microsatellite instability, positron emission tomography/computed tomography

## Text Correction

In the published article, there was an error. There was an error in the mathematical formula. We mistook Odds ratio to Regression coefficient.

A correction has been made to **Results**, to the first paragraph *Model establishment and mucinous composition exploration*. This sentence previously stated: x = -2.083 + 2.114 × Z-HI60%+ 13.32 × mucinous component (the “-” or “+” of mucinous component was defined as “0” or “l”).

The corrected sentence appears below: x = -2.083 + 0.749 × Z-HI60%+ 2.589 × mucinous component (the “-” or “+” of mucinous component was defined as “0” or “l”) (Table S3).

The authors apologize for this error and state that this does not change the scientific conclusions of the article in any way. The original article has been updated.

