# Supplementary material for: Predictive value of intratumoral-metabolic heterogeneity derived from 18F-FDG PET/CT in distinguishing microsatellite instability status of colorectal carcinoma
Source: Front Oncol. 2023 Apr 27;13:1065744. doi: 10.3389/fonc.2023.1065744 (PMC10173881; doi:10.3389/fonc.2023.1065744)
Supplement: Supplementary file 1 [file Table_1.docx]

Table S1: Correlations between PET relevant parameters and MSI status in patients with CRC

|  | **SUV_max_** | **SUV_mean_ with percent threshold method** | | | | | **MTV with percent threshold method** | | | | | **TLG with percent threshold method** | | | | | **HI with percent threshold method** | | | | | **HF** |
| --- | --- | --- | --- | --- | --- | --- | --- | --- | --- | --- | --- | --- | --- | --- | --- | --- | --- | --- | --- | --- | --- | --- |
|  |  | 30% | 40% | 50% | 60% | 70% | 30% | 40% | 50% | 60% | 70% | 30% | 40% | 50% | 60% | 70% | 30% | 40% | 50% | 60% | 70% |  |
| MSS | 14.56  (10.74,20.10) | 7.21  (5.43,9.71) | 8.10  (6.11,11.40) | 9.19  (7.06,13.0) | 10.24  (7.92,14.60) | 11.80  (8.86,16.30) | 16.63  (10.12,28.52) | 10.38  (6.57,18.19) | 6.80  (3.97,11.69) | 4.0  (2.31,6.84) | 1.95  (1.13,3.28) | 121.07  (65.40,244.23) | 94.71  (46.53,183.91) | 70.85  (31.43,119.78) | 44.90  (20.84,79.41) | 22.26  (11.31,42.76) | 2.02  (1.91,2.12) | 1.72  (1.66,1.8) | 1.53  (1.48,1.58) | 1.38  (1.35,1.41) | 1.25  (1.23,1.27) | 0.35  (0.21,0.63) |
| MSI-H | 16.45  (12.27,18.26) | 7.81  (6.12,8.80) | 8.86  (7.36,9.94) | 10.0  (8.11,11.20) | 11.21  (8.87,12.60) | 12.48  (9.72,14.10) | 24.0  (17.80,37.46) | 17.71  (11.70,26.91) | 9.71  (7.40,17.82) | 5.74  (3.44,10.18) | 2.73  (1.84,3.85) | 207.67  (135.74,295.47) | 161.43  (103.14,245.86) | 106.26  (62.52,177.32) | 63.33  (32.73,128.56) | 30.83  (13.94,64.38) | 2.01  (1.95,2.07) | 1.74  (1.71,1.84) | 1.56  (1.52,1.65) | 1.42  (1.39,1.45) | 1.27  (1.26,1.29) | 0.55  (0.42,0.83) |
| Z | -0.005 | -0.188 | -0.264 | -0.360 | -0.370 | -0.238 | -2.540 | -2.601 | -2.434 | -2.054 | -1.425 | -1.851 | -1.912 | -1.790 | -1.516 | -1.070 | -0.634 | -1.242 | -1.912 | -2.459 | -2.480 | -2.551 |
| *P* | 0.996 | 0.854 | 0.795 | 0.723 | 0.716 | 0.815 | 0.011 | 0.009 | 0.015 | 0.040 | 0.154 | 0.064 | 0.056 | 0.073 | 0.129 | 0.285 | 0.526 | 0.214 | 0.056 | 0.014 | 0.013 | 0.011 |

**Abbreviations:** MSI, microsatellite instability; MSS, microsatellite stability; SUV, standardized uptake value; MTV, metabolic tumor volume; TLG, total lesion glycolysis; HI, heterogeneity index; HF, heterogeneity factor
