# Supplementary material for: Predictive value of intratumoral-metabolic heterogeneity derived from 18F-FDG PET/CT in distinguishing microsatellite instability status of colorectal carcinoma
Source: Front Oncol. 2023 Apr 27;13:1065744. doi: 10.3389/fonc.2023.1065744 (PMC10173881; doi:10.3389/fonc.2023.1065744)
Supplement: Supplementary file 2 [file Table_2.docx]

Table S2: Correlations between PET relevant parameters and mucinous component in patients with CRC

|  | **SUV_max_** | **SUV_mean_ with percent threshold method** | | | | | **MTV with percent threshold method** | | | | | **TLG with percent threshold method** | | | | | **HI with percent threshold method** | | | | | **HF** |
| --- | --- | --- | --- | --- | --- | --- | --- | --- | --- | --- | --- | --- | --- | --- | --- | --- | --- | --- | --- | --- | --- | --- |
|  |  | 30% | 40% | 50% | 60% | 70% | 30% | 40% | 50% | 60% | 70% | 30% | 40% | 50% | 60% | 70% | 30% | 40% | 50% | 60% | 70% |  |
| mucinous (-) | 15.71  (11.18,19.90) | 7.45  (5.74,9.70) | 8.45  (6.65,11.25) | 9.50  (7.63,12.83) | 10.66  (8.27,14.50) | 12.30  (9.08,16.19) | 18.24  (10.66,30.03) | 12.22  (6.76,19.78) | 7.72  (4.24,12.10) | 4.24  (2.55,7.28) | 2.15  (1.24,3.70) | 145.57  (66.48,275.58) | 106.61  (48.36,214.19) | 75.22  (34.18,151.99) | 45.71  (23.62,101.12) | 22.71  (13.35,56.93) | 1.98  (1.91,2.08) | 1.71  (1.67,1.80) | 1.52  (1.48,1.59) | 1.38  (1.35,1.41) | 1.25  (1.23,1.27) | 0.39  (0.22,0.68) |
| mucinous (+) | 13.18  (8.14,18.15) | 6.48  (4.05,8.32) | 7.51  (4.76,9.45) | 8.57  (5.32,10.71) | 9.68  (5.92,12.27) | 10.64  (6.39,14.07) | 22.29  (16.12,30.86) | 13.57  (9.44,19.79) | 8.40  (5.55,10.38) | 4.13  (2.70,5.85) | 2.02  (0.93,2.84) | 141.92  (76.77,270.68) | 108.15  (54.84,187.70) | 75.02  (34.69,106.57) | 44.58  (19.35,64.31) | 24.07  (10.60,35.57) | 2.06  (2.0,2.17) | 1.77  (1.71,1.86) | 1.56  (1.53,1.62) | 1.40  (1.36,1.43) | 1.27  (1.23,1.28) | 0.48  (0.34,0.73) |
| Z | -0.952 | -1.223 | -1.131 | -1.104 | -1.087 | -0.968 | -0.876 | -0.40 | -0.016 | -0.519 | -0.974 | -0.097 | -0.270 | -0.563 | -0.757 | -1.071 | -2.034 | -1.807 | -1.558 | -1.049 | -1.039 | -0.855 |
| *P* | 0.346 | 0.225 | 0.262 | 0.274 | 0.281 | 0.337 | 0.386 | 0.694 | 0.989 | 0.609 | 0.335 | 0.927 | 0.793 | 0.581 | 0.456 | 0.290 | 0.042 | 0.071 | 1.121 | 0.299 | 0.305 | 0.398 |

**Abbreviations:** SUV, standardized uptake value; MTV, metabolic tumor volume; TLG, total lesion glycolysis; HI, heterogeneity index; HF, heterogeneity factor
