# Supplementary material for: Predictive value of intratumoral-metabolic heterogeneity derived from 18F-FDG PET/CT in distinguishing microsatellite instability status of colorectal carcinoma
Source: Front Oncol. 2023 Apr 27;13:1065744. doi: 10.3389/fonc.2023.1065744 (PMC10173881; doi:10.3389/fonc.2023.1065744)
Supplement: Supplementary file 3 [file Table_3.docx]

| Factors | Coefficient | Odds ratio | *P* value | 95% CI |
| --- | --- | --- | --- | --- |
| Mucinous component | 2.589 | 13.320 | <0.001 | 3.61-49.08 |
| Z-HI_60%_ | 0.749 | 2.114 | 0.037 | 1.05-4.28 |
| Constant | -2.083 | 0.125 | <0.001 |  |

Table S3 The two predictors of the logistic regression mathematical model for MSI

Z-, processed by Z-score standardization method; HI, heterogeneity index; CI, confidence interval.
